# Highly flexible cell membranes are the key to efficient production of lipophilic compounds

**DOI:** 10.1016/j.jlr.2024.100597

**Published:** 2024-07-17

**Authors:** Qiyao Zhu, Sijia Wang, Gang Fu, Fengming Guo, Wei Huang, Tengyue Zhang, Huina Dong, Zhaoxia Jin, Dawei Zhang

**Affiliations:** 1 School of Biological Engineering, Dalian Polytechnic University, Dalian, China; 2 Tianjin Institute of Industrial Biotechnology, Chinese Academy of Sciences, Tianjin, China; 3 Key Laboratory of Engineering Biology for Low-Carbon Manufacturing, Tianjin Institute of Industrial Biotechnology, Chinese Academy of Sciences, Tianjin, China; 4 National Center of Technology Innovation for Synthetic Biology, Tianjin, China

**Keywords:** cell membrane engineering, lipophilic compounds, synthetic biology, microbial cell factories, artificial storage compartments, product outflow

## Abstract

Lipophilic compounds have a variety of positive effects on human physiological functions and exhibit good effects in the prevention and treatment of clinical diseases. This has led to significant interest in the technical applications of synthetic biology for the production of lipophilic compounds. However, the strict selective permeability of the cell membrane and the hydrophobic nature of lipophilic compounds pose significant challenges to their production. During fermentation, lipophilic compounds tend to accumulate within cell membrane compartments rather than being secreted extracellularly. The toxic effects of excessive lipophilic compound accumulation can threaten cell viability, while the limited space within the cell membrane restricts further increases in production yield. Consequently, to achieve efficient production of lipophilic compounds, research is increasingly focused on constructing robust and multifunctional microbial cell factories. Utilizing membrane engineering techniques to construct highly flexible cell membranes is considered an effective strategy to break through the upper limit of lipophilic compound production. Currently, there are two main approaches to cell membrane modification: constructing artificial storage compartments for lipophilic compounds and engineering the cell membrane structure to facilitate product outflow. This review summarizes recent cell membrane engineering strategies applied in microbial cell factories for the production of liposoluble compounds, discussing the challenges and future prospects. These strategies enhance membrane flexibility and effectively promote the production of liposoluble compounds.

As the world faces the depletion of fossil fuel resources and increasing environmental degradation, there is new urgency to discover sustainable and eco-friendly methods for chemical production. Engineered microbial cell factories, with their potential to convert low-value renewable resources into high-value compounds, stand at the forefront as a promising alternative for chemical production (1). Lipophilic compounds are a class of substances carrying hydrophobic groups such as hydrocarbon and ester groups. Naturally lipophilic compounds produced through microbial fermentation are widely used in the fields of medicine, food, chemical engineering, and cosmetics. Among them, lipophilic compounds such as carotenoids, menaquinone-7, and lycopene are of particular interest due to their unique physiological functions in the human body (Fig. 1A) (2, 3, 4, 5).

**Fig. 1:**
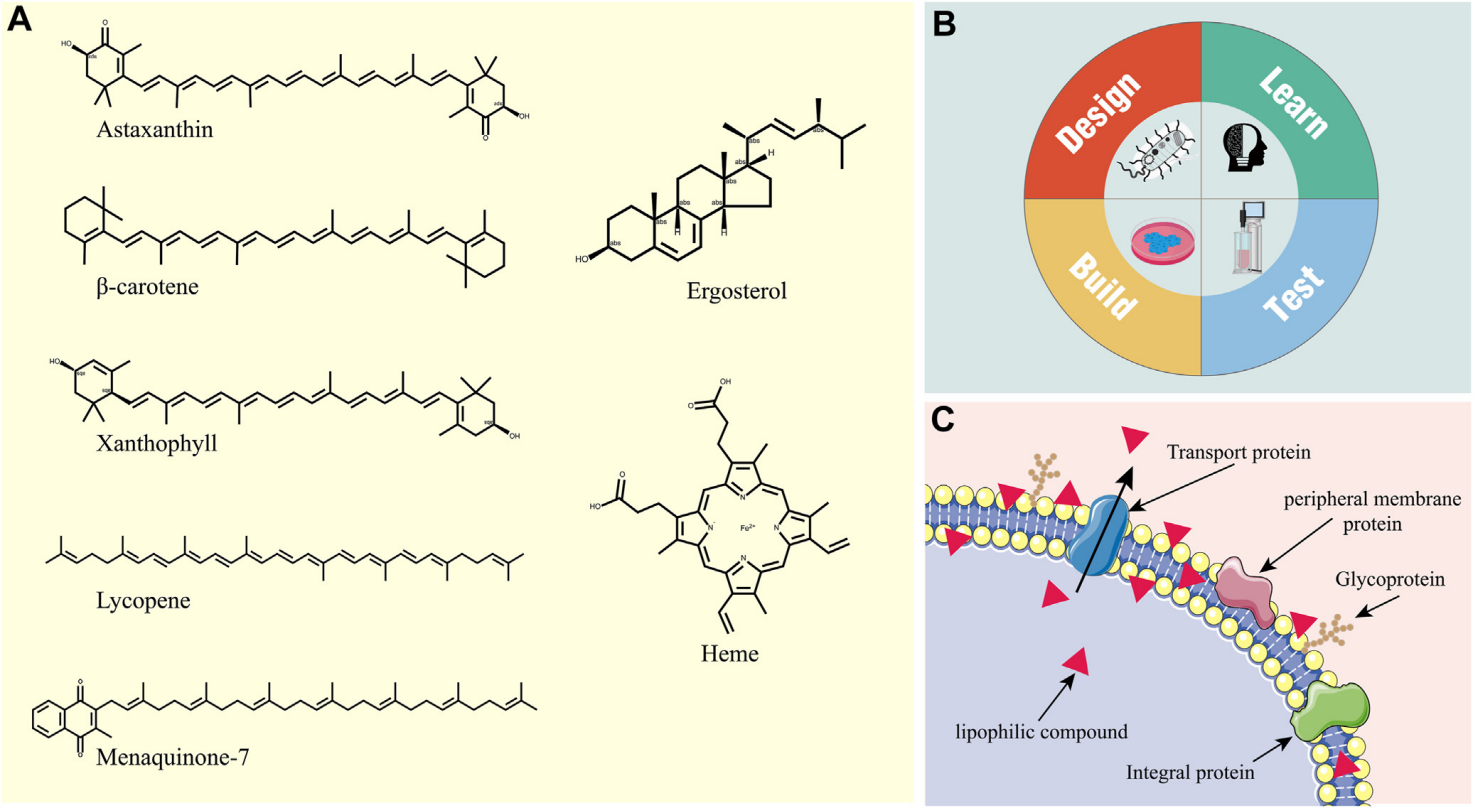
The application of membrane engineering technology in the production of lipophilic compounds. A: attractive lipophilic compounds. B: the DBTL (Design-Build-Test-Learn) cycle centered on metabolism serves as the core idea for the construction of microbial cell factories. C: cell membrane structure, lipophilic compounds (represented by red triangles) can be excreted from the cytoplasm by transporter proteins, but most accumulate in the phospholipid bilayer.

The hydrophobic groups of lipophilic compounds form hydrophobic interactions with the water environment, making it difficult to dissolve into water (6). Therefore, the production of lipophilic compounds using microbial fermentation may be challenging. The inherent inability of lipophilic compounds to traverse the cell membrane results in their aggregation in the cytoplasm as lipid clusters or accumulation within membrane compartments, which spatially restricts the production rate of lipophilic compounds. Despite attempts at enhancing metabolic pathways, this spatial limitation hinders the effective increase in yield (7).

Cellular membranes, a universal feature across a wide range of biological entities, function as the essential bridge between the internal cellular environment and the external world (8). They play an indispensable role in preserving normal cellular functions and structural integrity. As one of the most complex cellular organelles, the cell membrane's intact structure is essential for its functions. However, the membrane's fluidity grants it a high degree of structural plasticity, allowing changes in its composition and structure within a controlled range without causing membrane damage or cell death (9, 10). Utilizing membrane engineering techniques to modify the components and structure of the cell membrane can enhance its flexibility, offering a promising strategy to overcome the challenge of difficulty in exporting lipophilic compounds produced by microorganisms. In the field of synthetic biology, numerous studies have demonstrated the significant potential of membrane engineering in constructing robust and multifunctional microbial cell factories. These advancements include engineering membrane components to create artificial storage compartments for lipophilic compounds, incorporating surfactants to modify membrane permeability, engineering membrane transport proteins to facilitate product efflux, and forming membrane vesicles to enhance product export.

In this review, we begin by presenting the structural features of membranes and summarizing the significant research advancements in this domain. Subsequently, we provide a detailed discussion of the diverse physiological health benefits of lipophilic compounds, exemplified by carotenoids and menaquinone-7, and highlight the challenges faced by microbial cell factories in producing these compounds. Finally, by reviewing recent applications of cell membrane engineering techniques in biosynthesis, we emphasize that highly flexible cell membranes are the key to overcoming the limitations in the production of lipophilic compounds.

## The Structure of the Cell Membrane

Lipid molecules and proteins are interconnected through non-covalent interactions to form the cell membrane (11). Phospholipid molecules, which act as the foundational units of the cell membrane's architecture, account for the majority of membrane lipids. Each phospholipid molecule consists of a hydrophilic head group and two hydrophobic fatty acid tails. Through hydrophobic interactions and van der Waals forces, phospholipid molecules spontaneously organize into a compact phospholipid bilayer structure. This arrangement positions the hydrophobic tails inward and the hydrophilic heads outward, effectively segregating the cell’s internal environment from the external milieu (12). As shown in Fig. 1C, proteins interact with the phospholipid bilayer in various ways. Most membrane proteins, referred to as transmembrane proteins, are embedded in the phospholipid bilayer and perform diverse functions of the cell membrane, including the transport of specific molecules and the catalysis of membrane-associated reactions. In addition, the outer layer of the cell membrane also contains sugars covalently bound to lipids, forming glycoproteins. These glycoproteins, existing as polysaccharide chains of complete membrane protein polysaccharides, primarily reside outside the cell as part of the extracellular matrix. Glycoproteins play a crucial role in intercellular signal transmission, helping to maintain intercellular distances and prevent unnecessary protein interactions (13).

In the quest to understand the structure and function of the cell membrane, researchers have proposed various models to elucidate the relationship between the two. In 1972, S. J. Singer and Garth L. Nicolson introduced the fluid mosaic model, which characterizes the cell membrane structure as a fluid phospholipid bilayer that forms the fundamental framework of the membrane. Proteins can be embedded in, envelop, or penetrate the phospholipid bilayer (11). These proteins and phospholipids exhibit a degree of fluidity, rendering the membrane structure in a state of constant flux. Consequently, the structure of the cell membrane is mechanically fluid and functionally selectively permeable. Although the fluid mosaic model remains the leading theory for explaining the characteristics of the cell membrane, it is essential to acknowledge that the complex structure and dynamic properties of the membrane cannot be fully explained by a single model. Continual progress in nanotechnology and imaging technology, together with the use of advanced experimental tools and research methods like Atomic Force Microscopy (AFM) and Single Molecule Force Spectroscopy (SMFS), have enabled an in-depth exploration of various cell membrane structures (14), leading to revisions and supplements to the fluid mosaic model (8, 15, 16, 17). As more complex structures with the cell membrane are gradually revealed, the fluid mosaic model appears insufficient in providing theoretical support for deeper levels of cell membrane structure. Therefore, the raft hypothesis has once again attracted the attention of researchers. Unlike the fluid mosaic model, the raft hypothesis proposes that specific lipids in the cell membrane form lateral compartments, termed lipid rafts (18). The experimental results from super-resolution microscopy reveal the existence of raft structures in living cells, opening up a new realm for the study of cell membrane structure and function (19, 20). Lipid rafts, rich in cholesterol, myelin proteins, and membrane proteins, perform various functions on the cell membrane, including signal transduction, protein-mediated endocytosis, and viral infection (21, 22, 23). According to recent studies, the bacterial cell membrane also has raft structures identified as membrane-functional microdomains that are similar to those found in eukaryotic cells (24). Flotillin-like proteins are situated in the membrane functional microdomains and play a crucial role in recruiting proteins and maintaining FMM structure (25, 26). Furthermore, researchers have recognized that specific interactions among membrane proteins and between lipid domains may restrict the lateral movement of the cell membrane (16, 27). Numerous experimental findings indicate that transmembrane proteins cannot move freely within the plane of the cell membrane, and the presence of lipid domains further slows diffusion within the plasma membrane (28, 29). This suggests that the fluid dynamics of the lipid bilayer may not support free planar diffusion as originally described in the initial version of the fluid mosaic model. The 2D-gel model proposed by Adam *et al.* offers fresh insights into cell membrane dynamics. Their research findings show that the purified phospholipid bilayer, undisturbed by external forces, can flow on the membrane plane akin to honey in vitro. In cells, however, the obstruction of transmembrane proteins imparts the membrane with the properties of a semi-solid gel (29). This finding is significant for understanding how membrane tension activates ion channels and regulates the dynamic structure of the cell membrane. Regrettably, the role and potential impact of raft structures in the 2D-gel model have not been addressed.

## Challenges in the Production of Lipophilic Compounds

**Fig. 2:**
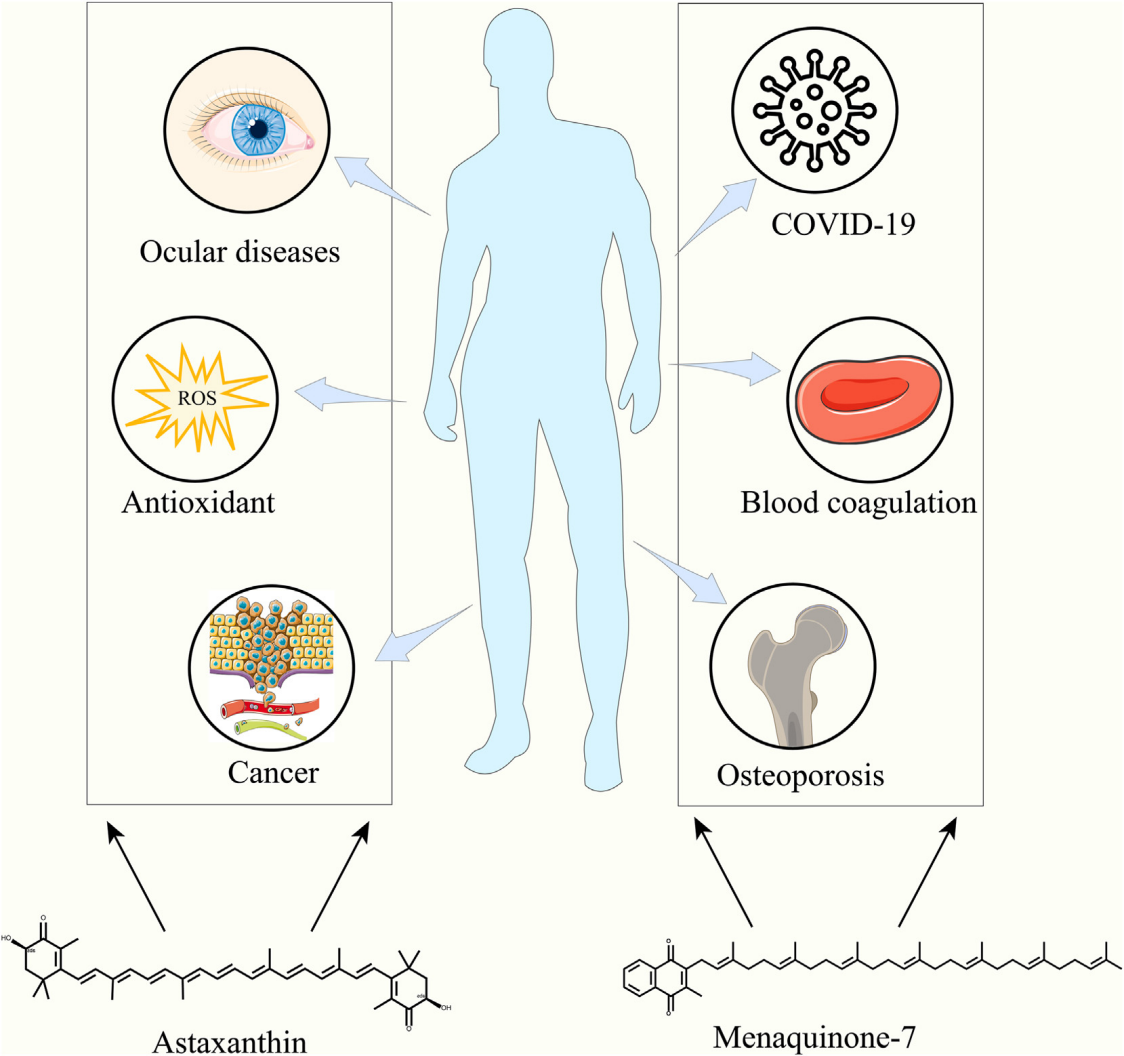
The physiological impacts of astaxanthin and menaquinone-7 on human health.

Lipophilic compounds, characterized by their hydrophobic groups, encompass a variety of chemical substances and typically tend to dissolve in lipids or nonpolar substances. Figure 1A presents the chemical structures of several lipophilic compounds that are of interest in the medical and health food sectors. Notably, natural lipophilic compounds such as carotenoids, menaquinone-7, and lycopene have garnered considerable attention due to their unique physiological benefits to human health. Carotenoids, exemplified by β-carotene, possess potent antioxidant properties, and their consumption has been associated with a reduced risk of cancer (30). Astaxanthin, another member of the carotenoid family, is commonly found within the flesh of a wide variety of fish species, as well as in the shells of crustaceans like shrimp and crabs (31). As shown in Fig. 2, Astaxanthin boasts an antioxidant activity that is tenfold greater than that of β-carotene, rendering it highly effective in the prevention and treatment of a variety of clinical conditions, including cancer, ocular diseases, atherosclerosis, and diabetes (32). Consequently, the development of microbial synthesis technology for astaxanthin is attracting increasing attention. Menaquinone-7 (MK-7) is a subtype of Vitamin K2, which has a higher bioavailability and a longer half-life in the human body (33). MK-7, gaining popularity as a dietary supplement, is highly valued for its efficacy in the prevention of osteoporosis and the promotion of blood coagulation (34). Recent studies have revealed that patients with COVID-19 exhibit significantly lower levels of MK-7 compared to healthy control subjects. Furthermore, it has been suggested that a deficiency of MK-7 within the human body may contribute to the exacerbation of COVID-19 symptoms (35, 36). Lycopene, recognized as a natural and edible pigment, is renowned for its robust antioxidant capabilities. Upon consumption, it can exert a beneficial influence at various stages of atherosclerosis progression (37).

Amid escalating concerns over various chronic diseases in recent years, there has been a surge in market demand for lipophilic compounds, which have demonstrated efficacy in the prevention and treatment of most chronic diseases. Consequently, the production of environmentally friendly and safe lipophilic compounds using microbial cell factories holds great promise. The design-build-test-learn (DBTL) cycle of synthetic biology methods has emerged as the primary strategy for constructing microbial cell factories (Fig. 1B) (38), significantly advancing the industrial production of lipophilic compounds. However, the spatial constraints of the cell membrane pose a challenge to the efficient production of these compounds. In the course of producing lipophilic compounds via microbial cell factories, these compounds exhibit a propensity to accumulate within the lipid-rich cell membrane, as opposed to being expelled from the cell. This suggests that the limited capacity of the cell membrane inherently dictates the maximum yield of lipophilic compounds, a perspective corroborated by pertinent literature (39). At the same time, it has been found that the accumulation of various lipophilic compounds in the compartments of the cell membrane can induce cytotoxicity and interfere with the normal function of the membrane (40, 41, 42). To solve this problem, current research focuses on using cell membrane engineering technology to adjust the components and structure of the microbial cell membrane to make the cell membrane more flexible, thereby providing more storage space for lipophilic compounds or helping them flow out from the cell membrane.

## Membrane Morphology Engineering Constructs Artificial Storage Compartments

The hydrophobic nature of lipophilic compounds, along with precise regulation by cell membranes for substance entry and exit, determines that these compounds cannot be easily expelled from the cell membrane. Instead, they accumulate on lipid-rich cell membranes or form hydrophobic lipid clusters within the cytoplasm. Regardless of the mode of aggregation, the substantial accumulation of lipophilic compounds disrupts normal cellular functions in microbial cell factories (43). Therefore, constructing artificial storage depots to overcome the capacity limitations imposed by cell membrane space for lipophilic compounds holds significant importance. A promising strategy involves enhancing the hydrophobic environment within cells to encapsulate lipophilic products. Both lipid engineering, which creates lipid droplets by enhancing endogenous lipid biosynthesis pathways within cells, and membrane engineering, which modifies the morphological characteristics of the cell membrane by regulating the expression of membrane morphology-related genes, have been extensively utilized in the field of biotechnology (7). The application methods of lipid engineering are beyond the scope of this review, and numerous excellent publications have summarized it (44, 45, 46). Here we discuss the potential of cell membrane morphological changes as a hydrophobic product storage warehouse and introduce the application cases of membrane engineering technology in the production of lipophilic compounds.

**Fig. 3:**
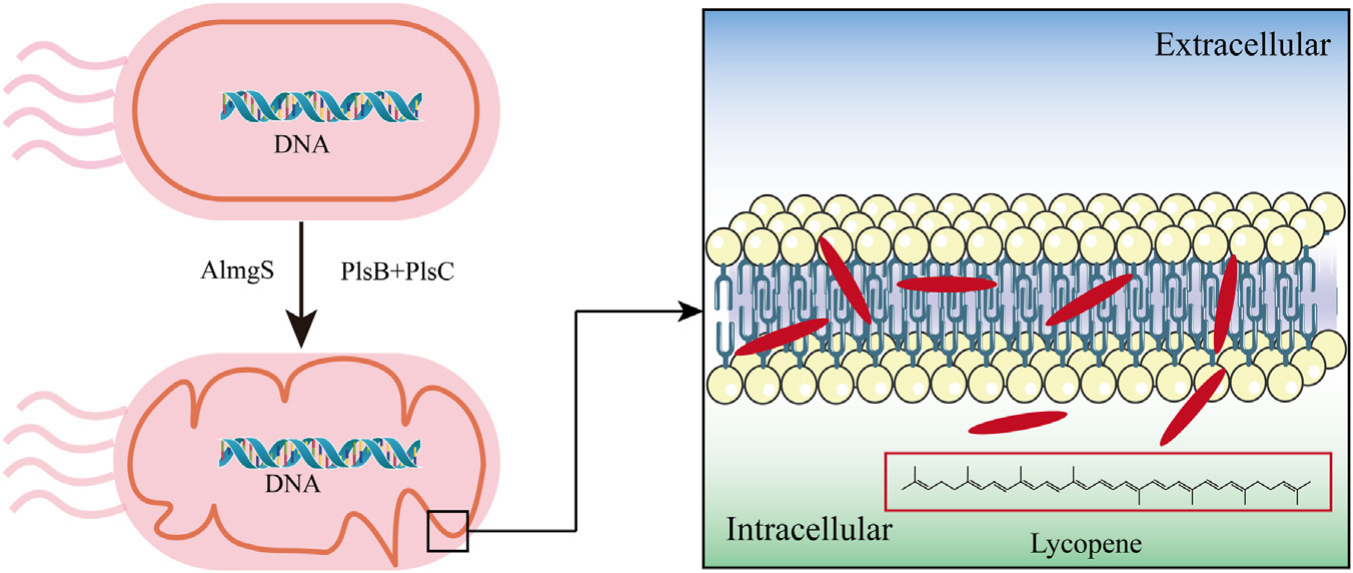
Cell membrane morphology engineering. The membrane-curving protein Almgs, derived from *Acholeplasma laidlawii*, is heterologously expressed in *E. coli*, causing the membrane morphology to become curved. PlsB, glycerol-3-phosphate-acyltransferase; PlsC, 1-acylglycerol-3-phosphate-acyltransferase (51).

Lipids are the primary hydrophobic components of the cell membrane (47). Increasing the quantity of hydrophobic components in the cell membrane is beneficial for the accumulation of lipophilic compounds. The biosynthesis of Diacylglycerol-3-P is enhanced by the over-expression of PlsB and PlsC, which provides an abundant precursor supply for the biosynthesis pathway of membrane components. This leads to an increase in the total amount of *E. coli* cell membrane components along with the enhanced production of β-carotene (48). Cell membrane curvature implies that cells have a larger surface area-to-volume ratio, providing increased storage space for the production of lipophilic compounds (49). Wu *et al.* heterologously expressed multiple genes that regulate cell membrane curvature in Escherichia coli, aiming to construct an artificial storage compartment for lipophilic compounds. These genes include the mannitol permease MtlA, the chemotactic receptor Tsr, and the membrane curvature protein Almgs. The curved membrane structure folds into a wrinkled shape, and the increased hydrophobic components provide more space for the storage of β-carotene, with the overexpression of the Almgs membrane curvature protein having the most significant effect on the increase in β-carotene production (50). As shown in Fig. 3, Wu *et al.* increased the production of lycopene by 12% by heterologously expressing the membrane curvature protein Almgs from *Acholeplasma laidlawii*. By overexpressing the genes involved in the *E.coli* cell membrane synthetic pathway, namely plsB, plsC, and dgkA, the total membrane content was increased. This expansion of the lipophilic compound storage space led to a further enhancement in lycopene production. Compared to the parental strain, the engineered strain with modified membrane morphology exhibited a 1.32-fold increase in lycopene yield (51). Cell membrane morphology engineering through heterologous expression of membrane curvature proteins has been demonstrated as an effective strategy to enhance the production of lipophilic compounds. However, the partial loss of membrane function and the potential impact on cellular activities caused by changes in cell membrane morphology and components cannot be ignored.

## Improving Cell Membrane Permeability to Alleviate Cytotoxicity

Bacteria have evolved a precise material transport system to tightly regulate the uptake of nutrients and the efflux of metabolic products. This relies on the phospholipid bilayer of the cell membrane and the embedded transport proteins within it (11). The phospholipid bilayer of the cell membrane separates the external environment from the cell’s interior. It allows small molecules like H_2_O and CO_2_ to pass freely, while larger compounds rely on transport proteins for transportation. During production, the cell membrane’s stringent selectivity hinders the efflux of lipophilic compounds, and allowing an excessive accumulation of these compounds can threaten the survival of host cells. Researchers are exploring various strategies to construct highly flexible cell membranes, aiming to continuously enhance lipophilic compound production and mitigate cellular toxicity.

Recent research has found that the permeability of the cell membrane changes after treatment with surfactants (52). Surfactant molecules, possessing amphiphilicity akin to phospholipid molecules, distribute into the membrane due to intermolecular forces. When a sufficient number of surfactant monomers aggregate in the cell membrane to form micelles, they dissolve phospholipid molecules, thereby increasing cell membrane permeability (53). Table 1 summarizes recent applications of surfactants to enhance cell membrane permeability. Surfactants such as Sodium Dodecyl Sulfate (SDS) and Triton X-100 can be used to dissolve the amphiphilic phospholipids on the cell membrane, facilitating the expulsion of metabolic products through the pores formed on the cell membrane (54). While enhancing cell membrane permeability, Tween-80 and Triton X-100 do not significantly affect cell morphology and function. Therefore, they are preferred surfactants for optimizing microbial fermentation. Cai *et al.* significantly enhanced the yield of hypocrellin to 780.6 mg/L in the fermentation process of *Shiraia sp.* SUPER-H168 by adding 0.6% Triton X-100. Simultaneously, it also reduces the fermentation duration (55). Another benefit of using surfactants during the biosynthesis of lipophilic compounds is to alleviate the accumulation of product in the cell membrane which could otherwise result in cytotoxicity. For example, lycopene is a lipophilic natural pigment, and the hydrophobic phospholipid molecular layer provides a storage space for this lipophilic compound. However, the limited cell membrane space and tolerance hinder further enhancement of lycopene production (5). Yoon *et al.* added 0.5% Tween-80 to the culture medium, which reduced the phenomenon of cell aggregation caused by the accumulation of lycopene in the cell membrane, while simultaneously promoting cell growth and the yield of lycopene (56). *Bacillus subtilis* serves as the primary chassis cell for the production of MK-7. However, the accumulation of MK-7 in the cell membrane leads to superoxide cytotoxicity, which impedes normal cellular activities (40, 57). To facilitate the release of MK-7 from the cell membrane into the fermentation broth, Zhou *et al.* attempted to add various types of surfactants to the culture medium, including cationic surfactant CTAB, anionic surfactant Sodium Dodecyl Sulfate, non-ionic surfactant Tween-80, Polyethylene Glycol-200, and amphoteric surfactants such as Diammonium Citrate and Betaine. The results demonstrated that the addition of 0.7% Tween-80 significantly increased the yield of extracellular MK-7, boosting the total yield of MK-7 by 80.3%. Scanning electron microscopy and flow cytometry results confirmed that the mixed micelles formed by the dissolution of phospholipid molecules and surfactant molecules altered the cell membrane structure, thereby facilitating the excretion of MK-7 outside the cell (58). Furthermore, Yi *et al.* added the non-ionic surfactant Brij-58 to the culture medium of *Bacillus subtilis natto*, which increased the total yield and secretion rate of MK-7 by 48.0% and 56.2% respectively. Brij-58 molecules adhere to the cell surface through van der Waals forces, hydrogen bonding, and hydrophobic effects, reducing the surface tension between the cell surface and the fermentation broth, increasing the fluidity of the cell membrane while reducing the integrity of the cell membrane structure, thereby leading to an increase in cell membrane permeability. This strategy provides a new research direction for the optimization of the MK-7 fermentation process (59). While adding surfactants to the fermentation broth may increase the workload for product purification, judicious use of surfactants can enhance the efficient production of lipophilic compounds. As a key component of chemical cleaning agents, surfactants are cost-effective and readily available, making them promising candidates for large-scale application in the industrial production of lipophilic compounds.

**Table 1:** The application of surfactants in the fermentation process of microbial cell factories

Surfactants	Chassis cell	Product	Production	References
Triton X-100	*Shiraia sp.* SUPER-H168	hypocrellin	Optimal supplementation at the onset of fermentation results in a yield of 780.6 mg/L;	(36)
Tween 80	*Escherichia coli*	Lycopene	Upon the incorporation of 0.5% Tween 80 into the culture medium, the yield escalated to 102 mg/L, concurrently facilitating the prevention of agglomeration;	(37)
Tween 80	*Bacillus subtilis*	Menaquinone-7	Upon the incorporation of 0.7% Tween-80, the intracellular and extracellular productivities escalated to 28.8 mg/L and 59.2 mg/L respectively;	(40)
Brij-58	*Bacillus natto*	Menaquinone-7	The overall productivity and secretion rate witnessed respective enhancements of 48% and 56.2%;	(41)
Tween 80	*Aspergillus nidulans*	Echinocandin B	Supplementation of Tween 80 in the culture medium enhances the yield to 2,584 mg/L;	(42)
Sodium Dodecyl Sulfate	*Corynebacterium glutamicum*	L-isoleucine	The yield escalated to 43.67 g/L, marking a 13.01% enhancement relative to the control, concurrently effectuating a significant reduction in by-product formation;	(43)
Triton X-100	*E. coli* BL21	Truncated recombinant mannanases	Incorporation of 0.1% Triton X-100 culminated in a total yield of 9,284.64 U/ml, with an extracellular productivity reaching 74%;	(44)
Tween 80 and Sodium Lauryl Sulfate	*Bacillus subtilis* BI19	Amylase	The supplementation of Tween 80 and sodium lauryl sulfate led to respective enhancements in enzyme yield by 28% and 15%;	(45)
Polyoxyethyleneoleyl	*Flavobacterium* sp. M1-14	Vitamin K2	The yield escalated to 25.55 mg/L, marking a 252.4% enhancement relative to the control;	(46)
Tween 80	*Bacillus subtilis*	Levan	Upon the incorporation of 0.5 g/L Tween 80, the yield peaked at 48.8 g/L.	(47)

## Membrane Transport Protein Engineering

Membrane transport proteins regulate the transport process of substances through facilitated diffusion. Most membrane transport proteins possess specific receptors, allowing only particular compounds to freely traverse the cell membrane (60). For systematic research, biological data from 15,228 distinct membrane transport systems across various organisms are cataloged in the Transporter Classification Database (TCDB, https://www.tcdb.org/), which researchers can access free of charge. A rigorous classification system has been established based on the functional and structural characteristics of transport proteins (61, 62, 63). The emergence of The Transporter Classification Database has opened up vast opportunities for membrane transport system innovation within the synthetic biology community.

Fatty acids are essential for the cytoplasmic membrane and are used extensively in the chemical and fuel sectors. When they build up in *E. coli*, however, they can induce stress responses and cell lysis (64). According to experimental findings, fatty acids are excreted from cells through a transcellular enveloping transport mechanism, with the AcrAB-TolC efflux pump performing the majority of this role (65). Wu *et al.* further demonstrated the importance of the AcrAB-TolC efflux pump for fatty acid production. By overexpressing the resistance-nodulation-cell division (RND) family transporter acrE, mdtE, and mdtC, the yield of medium-chain fatty acids increased by 46.4%, 65.2%, and 33.8% respectively. After overexpressing acrE, mdtE, and mdtC in combination and knocking out the multidrug efflux pumps cmr, the yield of medium-chain fatty acids increased more than twice (66). Heterologous expression of transporter proteins has also been proven to be an effective strategy to promote product efflux. Chen *et al.* expressed the heterologous ABC transport protein of *Yarrowia lipolytica* into *Saccharomyces cerevisiae*, which significantly increased the tolerance to undecane and tridecane (67). In addition, for compounds with clear membrane transport mechanisms, the application of membrane transport protein engineering can be more flexible. For instance, knocking out the heme precursor transporter genes cysG, hemX, and cyoE reduces the loss of heme precursors, which helps to boost the flux of heme biosynthesis. Overexpressing the heme extracellular transport genes dpp and ccm effectively increases the yield of heme (68). In another study, overexpression of the ABC transport protein permease YcjP, identified through transcriptomics, significantly enhanced *E. coli*’s ability to produce caffeic acid (69). Membrane transport protein engineering offers an effective solution to address the accumulation of lipophilic compounds. However, the complex process of identifying and elucidating membrane transport mechanisms limits research progress. As a result, the application scope of membrane transport protein engineering in constructing microbial cell factories remains quite limited. The use of transcriptomics to mine potential transport proteins can effectively improve the efficiency of membrane transport protein engineering.

## Engineering Membrane Vesicles Facilitates Product Efflux

**Fig. 4:**
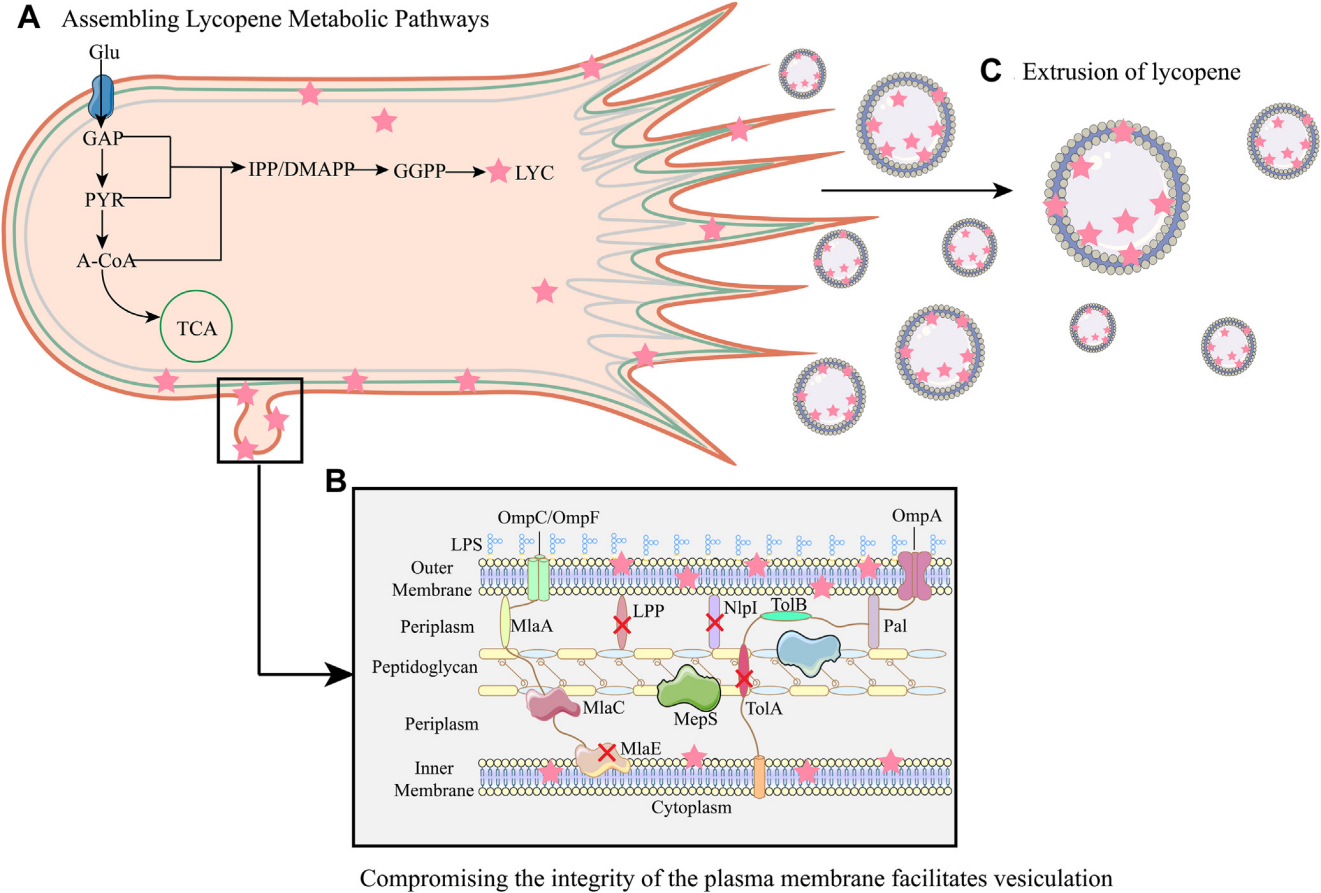
Membrane vesicles are utilized in the extrusion process of lycopene. A: In microbial cell factories, the lycopene biosynthesis pathway is assembled to enhance metabolic flux and promote lycopene production. B: By knocking out membrane-stabilizing proteins, the integrity of the outer membrane is disrupted, thereby inducing the production of membrane vesicles. C: Lycopene is excreted extracellularly along with the membrane vesicles. LPP, murein lipoprotein; MepS, murein DD-endopeptidase; MlaA/MlaC/MlaE, membrane lipid asymmetry pathway; NlpI, lipoprotein nlpI precursor; OmpC/OmpF/OmpA, outer membrane proteins; TolAB-Pal, Tol-Pal pathway (74).

Bacterial membrane vesicles were first discovered in *E*. *coli* (70). The controlled bubbling of the outer membrane of Gram-negative bacteria or cell lysis mediated by endolysins leads to the formation of membrane vesicles (71). These vesicles participate in various cellular functions, including cell communication, component modification, toxin transport, and DNA transfer (71, 72). The specific composition of the contents of membrane vesicles cannot be determined, but all types of membrane vesicles that have been discovered contain lipids, membrane proteins, lipoproteins, and a few soluble proteins attached to the periphery (72). Due to their unique transport and occurrence mechanisms, the application of membrane vesicles in the field of biotechnology is becoming increasingly widespread (73). For lipophilic compounds, membrane vesicles serve as natural transport carriers. When cellular membranes are perturbed to form vesicles, lipophilic compounds that remain encapsulated on the cell membrane are transported outside the cell along with the vesicles. Wu *et al.* disrupted the integrity of the outer membrane of *E. coli* by knocking out the outer membrane lipoprotein gene, which made membrane vesicles more likely to occur. The β-carotene accumulated on the membrane was transported to the culture medium with the membrane vesicles. At the same time, to prevent excessive loss of cell membrane components, the synthesis pathway of cell membrane components was enhanced, leading to a 24-fold increase in the extracellular production of β-carotene (48). As shown in Fig. 4, Eric Fordjour *et al.* disrupted the integrity of the outer membrane by knocking out murein lipoprotein LPP, lipoprotein NlpI, inner membrane permease protein MlaE, and membrane-anchored protein in E. coli TolA-TolQ-TolR, making the cells more prone to vesicle formation. At the same time, it was found that the production of membrane vesicles significantly increased the unsaturated fatty acid content of *E. coli*, thereby increasing the permeability of the cell membrane. By adding 20% dodecane to the culture medium for in situ extraction, the intracellular and extracellular production of lycopene was significantly increased. In addition, different mutant strains of the membrane protein gene tolA produced membrane vesicles of different sizes, and strains with reduced cell size showed a higher release rate of membrane vesicles. However, it is regrettable that current data cannot summarize the correlation between the number of membrane vesicles and the secretion rate of lycopene. The increase in extracellular lycopene production is believed to be due to the increased permeability and fluidity of the membrane (74). On the other hand, Mara Reifenrath used the Zera domain to induce the formation of artificial vesicles, which have a different formation mechanism from traditional membrane vesicles. These are anchored to the endoplasmic reticulum of *S. cerevisiae* and do not fall off, serving as a target for enzyme compartmentalization in the synthesis pathway, which is conducive to the aggregation effect of enzymes (75).

Gram-positive bacteria have a dense cell wall structure, so researchers have long overlooked the possibility of the occurrence of membrane vesicle mechanisms in Gram-positive bacteria. Recent research shows that Gram-positive bacteria also exhibit phenomena of membrane vesicle formation caused by bacterial autolysis (76, 77, 78). Unlike the formation of membrane vesicles in Gram-negative bacteria, the autolysins of Gram-positive bacteria cannot completely dissolve the cell wall, leading to the formation of membrane vesicles after the cell membrane ruptures, which cannot be expelled outside the cell wall. This mechanism of membrane vesicle formation is referred to as bubbling cell death (72). This also implies that membrane vesicles have the potential to be applied in cell membrane engineering technology in Gram-positive bacteria.

## Conclusion and Outlook

Recent advances in membrane engineering have positioned it as a potent strategy for creating multifunctional microbial cell factories specifically tailored for lipophilic compound production. This review highlights the crucial role of cell membranes in the production of lipophilic compounds. To mitigate the toxic effects of excessive lipophilic compound accumulation on the cell membrane, constructing highly flexible cell membranes through membrane engineering technology is essential for efficient production. However, as one of the most complex organelles in the cell, engineering membrane components and structures without interfering with the normal function of the cell membrane is challenging. The progress of membrane engineering technology is currently hindered by the lack of experimental evidence supporting theoretical models of the cell membrane (79).

With the continuous advancement of artificial intelligence technology in the field of biomedicine, computer-assisted scientific research will provide a more comprehensive blueprint for the construction process of highly flexible cell membranes. AlphaFold2, utilizing artificial intelligence technology, is poised to accelerate the research process of membrane proteins (80, 81). Moreover, the extensive use of machine learning platforms and generative artificial intelligence, combined with high-throughput instruments, can effectively reduce laboratory workload (82, 83). In the coming years, products powered by artificial intelligence will offer immense opportunities for the progression of membrane engineering technology. These products will contribute to the advancement of fundamental research in cell membranes and the development of synthetic biology technologies driven by machine learning platforms.

## Data Availability

All data are contained within the manuscript. All reagents are available upon request from the authors.

## Conflict of interest

The authors declare that they have no conflicts of interest with the contents of this article.
